# Magnetic Resonance Visibility, Artifacts, and Overall Safety of the Self-Locating Peritoneal Dialysis Catheter with a Tungsten Tip

**DOI:** 10.1155/2023/7901413

**Published:** 2023-01-24

**Authors:** Maurizio Gallieni, Umberto G. Rossi, Massimo Tonolini, Andrea Ianniello, Roberta Miglio, Gianmarco Sabiu, Maurizio Cariati

**Affiliations:** ^1^Nephrology and Dialysis Unit, ASST Fatebenefratelli Sacco, Milano, Italy; ^2^School of Nephrology, Università di Milano, Milano, Italy; ^3^Department of Biomedical and Clinical Sciences, Università di Milano, Milano, Italy; ^4^Department of Radiological Area, Interventional Radiology Unit, Galliera Hospital, Genova, Italy; ^5^Department of Radiology, ASST Fatebenefratelli Sacco, Milano, Italy; ^6^Transplantation Research Center, Brigham and Women's Hospital, Harvard Medical School, Boston, USA; ^7^Diagnostic and Interventional Radiology Unit, Department of Diagnostic and Therapeutic Advanced Technology, ASST Santi Paolo and Carlo, Milano, Italy

## Abstract

**Background:**

The self-locating peritoneal dialysis (PD) catheter, contains a tungsten tip. The effects of magnetic resonance (MR) on the catheter were evaluated, emphasizing its MR signal, artifacts, ferromagnetism, and possible heating production during the MR sequences.

**Methods:**

The catheter was studied in an ex vivo model using a 1.5T MR system and placed into a plastic box containing saline solution. Acquisitions on coronal and axial planes were obtained on fast gradient-echo T1-weighted and fast spin-echo T2-weighted. In vivo abdominal MR exams were also carried out.

**Results:**

Overall, the catheter had good visibility. In all sequences, an extensive paramagnetic blooming artifact was detected at the level of the tip tungsten ballast, with a circular artifact of 5 cm in diameter. The catheter showed no magnetic deflection, rotation, or movements during all MR sequences. After imaging, the temperature of the saline solution did not change compared to the basal measurement. Patients safely underwent abdominal MR.

**Conclusions:**

The results point to the possibility of safely performing MR in PD patients carrying the self-locating catheter. The self-locating PD catheter is stable when subjected to a 1.5T MR system. However, it creates some visual interference, preventing an accurate study of the tissues surrounding the tungsten tip.

## 1. Introduction

The self-locating peritoneal dialysis (PD) catheter was designed to avoid dislocation [1, 2]. It is a classic straight Tenckhoff catheter with the usual internal diameter of 2.6 mm, modified with a heavy tip built with 12 grams of silicone-encased tungsten, which is physically and chemically inert. The heavier catheter tip stays in the lower abdomen, while the classic Tenckhoff catheter usually floats on top of the dialysis fluid, carrying a higher risk of dislocation. In addition, if the self-locating PD catheter is displaced for any reason (e.g., as in a patient who kept an upside-down position), it will return to the lower abdomen after walking for a short time [3].

Tungsten is a paramagnetic element like aluminum, oxygen, titanium, and iron oxide. Studies on the behavior of paramagnetic elements show that magnetic fields do not influence them. On the contrary, paramagnetic elements can interfere with the magnetic field surrounding them, leading MRI systems to artifacts in the acquired images [4]. Indeed, in the product information of the self-locating catheter, the following sentence is reported: *“The catheter ballast may interfere with MR diagnostic techniques.”* Thus, it is not clear how the catheter interferes with the magnetic field, reducing the visibility of the surrounding structures, or if it can be harmful to the patient.

This study aimed to evaluate the behavior of the self-locating PD catheter when subjected to MR, emphasizing its MR signal, artifacts, ferromagnetism, and possible heating production during the MR sequences.

## 2. Materials and Methods

**Figure 1:**
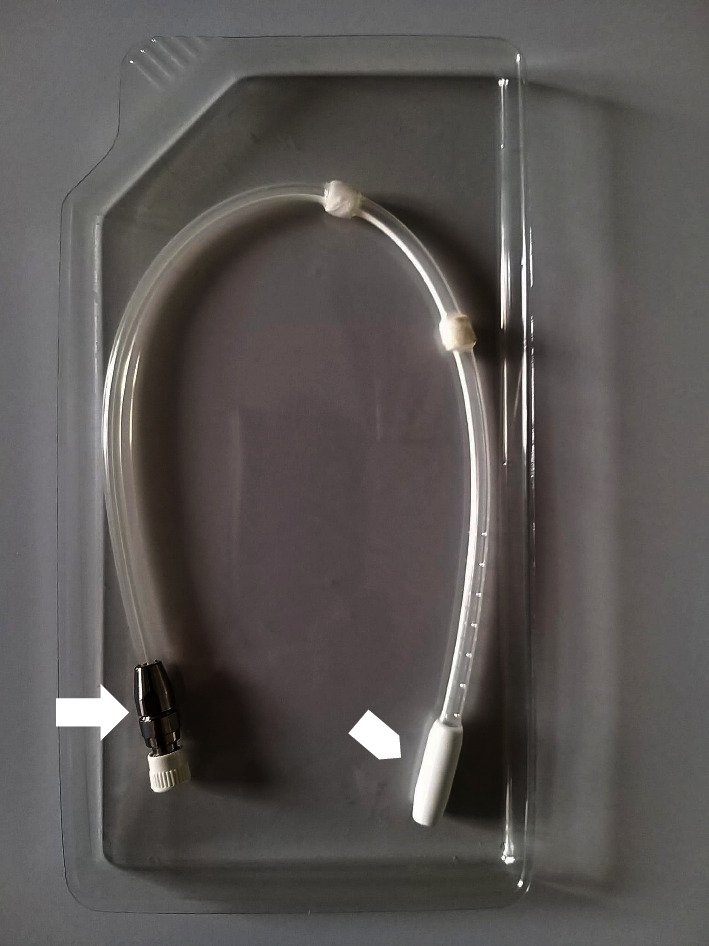
Peritoneal dialysis catheter into the plastic box containing saline solution (^*∗*^) with its tip tungsten ballast (arrowhead) and, on the other extremity, the titanium connector (arrow).

**Figure 2:**
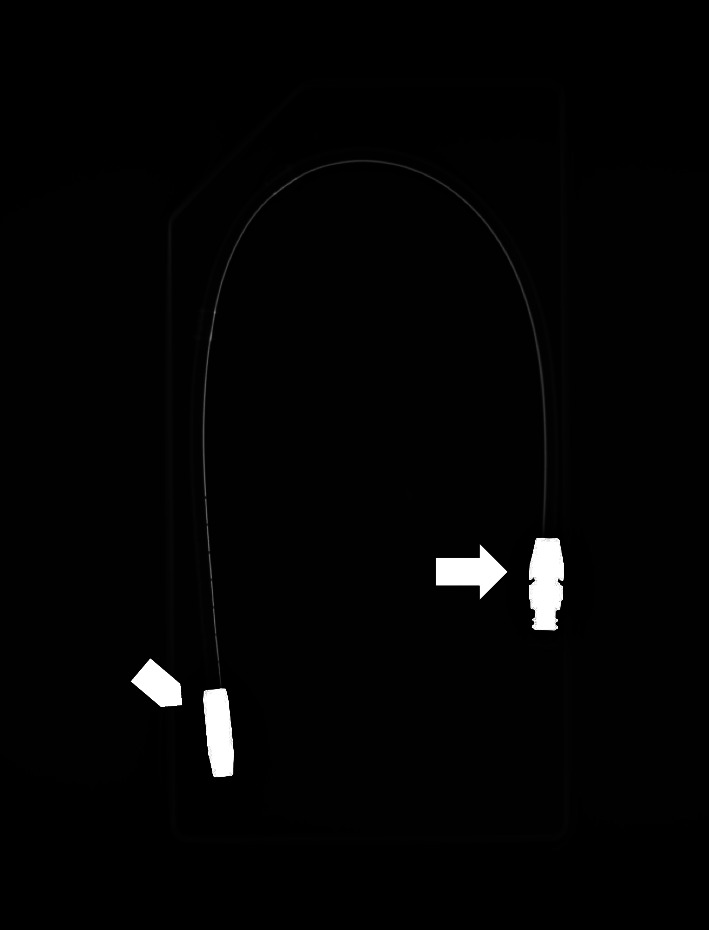
Conventional X-ray of the peritoneal dialysis catheter that demonstrates the radiopaque tip tungsten ballast (arrowhead) and, on the other extremity, the titanium connector (arrow).

**Figure 3:**
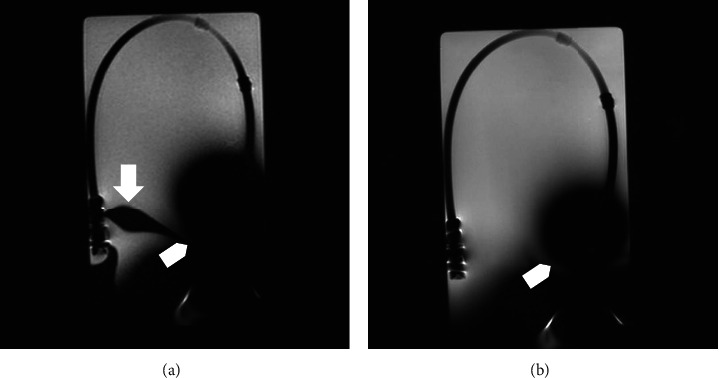
(a) Coronal fast gradient-echo T1-weighted and (b) coronal fast spin-echo T2-weighted images that demonstrate the extensive paramagnetic artifact with the “signal hole” of the tip tungsten ballast (arrowhead), the minor artifacts of the titanium connector (arrow), and the good visibility of the catheter.

A GoldSeal 1.5T Signa Excite HD magnetic resonance imaging system (General Electric Healthcare, Little Chalfont, United Kingdom) of 1.5 Tesla (T) was used to evaluate the self-locating PD catheter (Care-Cath: B. Braun Avitum Italy, Mirandola, Italy). The catheter was tested after attachment at the external extremity of a locking adapter titanium connector (Baxter Healthcare Inc., Deerfield, Illinois, USA) commonly used in clinical practice. Thus, both tungsten (at the catheter tip) and titanium were subjected to the MR field. The titanium-loaded, self-locating PD catheter was placed into a plastic box containing 150 ml of saline solution (NaCl, 0.9%), and it was oriented in parallel with the MR detector (Figure 1).  Figure 2 shows the standard X-ray appearance of the self-locating PD catheter used in this experiment. Acquisitions on coronal planes (Figure 3) were obtained on 3-mm thick fast spin-echo T1-weighted (TR/TE 400/10 msec) and fast spin-echo T2-weighted (TR/TE 3000/90 msec) sequences, using a 16-channel body array surface coil. The total acquisition time of the two MR sequences was 8 minutes. Catheter signal and displacement, size of the artifacts, and possible heating production were evaluated under the same conditions by two experienced radiologists. The heating was evaluated by measuring the temperature of the saline solution in which the self-locating PD catheter was immersed for the test. The temperature was measured immediately before and immediately after imaging with a digital thermometer sensitive to 0.1°C.

We also report representative MR images of the abdomen of patients who underwent MR exams.

## 3. Results

In all MR sequences (Figure 3), an extensive paramagnetic blooming artifact (a “signal hole”) was demonstrated at the level of the tip tungsten ballast, with a circular artifact of about 5 cm in diameter (Figure 3). The titanium connector generated minor artifacts (<1.5 cm), and overall the catheter had good visibility.

Catheter safety-wise, when placed into the magnetic field, the peritoneal dialysis catheter showed no magnetic deflection. Moreover, there was no evidence of rotation or movement during all MR sequences. No difference in temperature of the saline solution was detected immediately before and immediately after imaging; the temperature remained stable at 17.3°C.

Following these findings, we obtained informed consent to perform abdominal MR scans on PD patients with weighted catheters. Three patients with a self-locating catheter underwent MR scans of the abdomen: one for a study of the biliary tract and two for the dorsal and lumbar spines, respectively.

**Figure 4:**
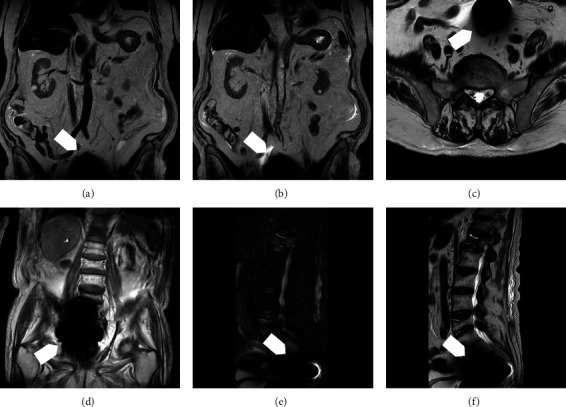
(a) Coronal fast-spin echo T1- and (b) T2-weighted images from an unenhanced abdominal MR study showing extensive paramagnetic “blooming” artifact at the lower end (arrow), corresponding to the site of the tip tungsten ballast. Axial T2- (c), coronal T1- (d), sagittal fat-suppressed (e), and T2- (f) weighted images performed to assess osteoporotic vertebral collapses show paramagnetic artifact (arrows) partially obscuring the anterior aspect of the sacrum.

Although a significant artifact surrounded the tungsten tip in pelvic projections, affecting the visibility of the lower lumbar spine and sacrum (Figure 4(c)), the self-locating catheter was confirmed to be safe, with the patients not suffering from any side effects. Moreover, the abdomen, the retroperitoneal area, the upper lumbar region, and the dorsal spine scan were not affected by the artifact produced by the tungsten tip (Figures 4(a)–4(f)).

## 4. Discussion

The self-locating PD catheter has the advantage of reducing dislocation from the lower abdomen. Several observational studies [5, 6] and two randomized control trials [7, 8] proved this feature. Complications such as cuff extrusion, tunnel infection, PD-associated peritonitis, early leakage, and obstruction were also statistically less frequent in patients with self-locating catheters than in those with classic Tenckhoff catheters. In addition, the reduced malfunction rate of the self-locating catheter is associated with a lower chronic laxative burden among PD patients [9].

However, concerns have been raised regarding the performance of an MR exam in patients carrying the self-locating PD catheter because tungsten is a high-density rare metal, although chemically inert. In addition, the possible presence of ferrous impurities in the tungsten catheter tip has been hypothesized. For these reasons, despite the lack of reliable epidemiological studies on its real diffusion, the use of the self-locating catheter seems to be limited worldwide. Therefore, one of the study's main aims was to dispel any doubts about the safety of the catheter in order to implement its use.

Although several studies on MR peritoneography have previously shown the safety and accuracy of MR in studying catheter-related complications in PD patients, none of them focused on self-locating catheters [10–12]. A recent study showed a case series of 14 PD patients who underwent MR for several reasons, mainly a central nervous system study. Notably, none of them underwent abdominal MR imaging [13]. Similar to our results, it showed a good safety profile of the self-locating catheter in the clinical setting and no deflection in the ex vivo experiments. The self-locating catheter demonstrated MR interference with the appearance of artifacts in both T1 and T2 around the catheter tip, with a greater extension with the 3T MRI (about 12 cm) than with the 1.5T MRI (about 8 cm). Only the 1.5T MRI was tested in our study, showing interference of about 5 cm. As previously reported, a more prominent interference is expected with a 3T MR machine [14]. Adding to the published ex vivo data, we confirmed that the tungsten-containing self-locating catheter produces artifacts in vivo with the 1.5T MRI.

Metallic biomedical implants and devices lead to artifacts in the magnetic resonance during patient scanning. Ferromagnetic materials are contraindicated for magnetic resonance because these materials are set into motion during magnetic resonance scanning [15]. The following unwanted events may occur with ferromagnetic materials: attractive effects and resultant patient injury; radiofrequency interference with the MR imaging study and secondary image artifacts; radiofrequency power deposition leading to device heating and secondary patient injury [16, 17].

Even with nonferromagnetic metals, such as titanium, the presence of metallic implants inside the body can determine MR susceptibility artifacts [18] and radiofrequency (RF) overflow artifacts [15, 19], causing a nonuniform appearance to an image due to perturbation of RF homogeneity near the metal part. In addition, the electric conductivity of the metal allows the high-frequency electromagnetic fields during an RF pulse to induce electric currents. Thus, a metal object may prevent the RF field from passing into a tissue, causing a signal void in an image close to the metal object. Interestingly, an in vitro study [14] investigating the RF-metal interaction effects caused by metallic instruments and implants made of titanium or nitinol (biopsy needles, hip prostheses, vascular stents, and aneurysm clips) showed that interference is more prominent at 3T.

In the present study, we tested at 1.5T the presence of interference and instability of the self-locating peritoneal catheter carrying a tungsten tip intended to be inserted in the peritoneal cavity and the external titanium connector.

Tungsten, the chemical element with the symbol W for Wolfram and a density of 19.3 g·cm^−3^, is a hard, rare metal. In other words, its density is 19.3 times that of water, comparable to that of gold, and significantly higher than that of lead and titanium. Metallic tungsten is hypoallergenic. Its conductive properties made it one of the primary sources for X-ray [20]. Compared to nonmagnetic materials with a relative permeability of 1, tungsten is paramagnetic with a relative permeability of 1.000068 [21]. Thus, tungsten-based devices should be MR safe, although they might show signal interferences. In addition, the nonhomogeneous composition of the metal tungsten tip could also be a source of MR interference artifacts. A previous study [22], performed in the neuro-interventional field, tested the MR safety of endovascular microcatheters with nitinol, tungsten, and polyetheretherketone braiding at 1.5T and 3T because of the possibility that microcatheter fragments may be entrapped in patients following endovascular procedures. Subsequent diagnostic MR examinations thus pose a safety concern due to the possibility of radiofrequency heating of the metallic braid incorporated into the microcatheter. In contrast to nitinol microcatheters, the tungsten braided microcatheters did not demonstrate heating and therefore showed potential for safe use in MR imaging.

We demonstrated that the tungsten tip ballast generates signal artifacts that, even when large, do not impair the assessment of the upper abdominal organs and upper spine. However, evaluation of the pelvic structures, such as the genital organs, rectum, and sacral spine, would be critical. In addition, we showed that no movement of the catheter or generation of heat was observed at 1.5T. Moreover, to our knowledge, this is the first study to show the effects of abdominal MRI exams in PD patients with a self-locating catheter, confirming the presence of local artifacts shown ex vivo in previous publications [13] and in our experimental setting but demonstrating the absence of detectable side effects in vivo. However, until more extensive experience is available in humans, during MR imaging in patients with the self-locating catheter, we suggest filling the peritoneal cavity with 0.5 liters of PD fluid, as we did in our patients, to 2 liters, as is usually done in MR peritoneography. The fluid will disperse any heat produced by the catheter tip, thus decreasing the already very low risk of a clinically significant thermal injury to adjacent tissues.

The present study has some limitations. First of all, the ex vivo experiments and in vivo analyses were performed only with a 1.5T MRI because it was the only one available to us, whereas higher intensity MRIs are commercially available (3, 4, 5, or even 8 Tesla). Only by testing the catheter with these more potent MRI scanners will it be possible to understand if the self-locating catheter will confirm its compatibility. We suggest testing the catheter ex vivo, as we did in our study, before using a 3T or higher MRI machine on a patient carrying this kind of catheter. In addition, as a single-center pilot study, only three patients were accurately studied in the in vivo analysis. However, 12 additional patients with the tungsten-containing self-locating PD catheter underwent MRI imaging of the central nervous system or the abdomen without clinical consequences. Future studies with larger case series and higher-intensity MRI are needed to confirm our findings.

In conclusion, this study demonstrates that the self-locating PD catheter is reasonably safe when subjected to a 1.5 Tesla MR system, although it creates some visual interference, preventing an accurate study of the tissues surrounding the tungsten tip. These findings point to the possibility of performing MR imaging studies in PD patients carrying the self-locating catheter.

## Data Availability

The data used to support the findings of this study are included within the article.
